# A matched case-control study on polypharmacy and co-medications one year before drug treatment for Alzheimer's disease

**DOI:** 10.1177/13872877241305799

**Published:** 2025-01-10

**Authors:** Ali Alghamdi, Stijn de Vos, Jens HJ Bos, Catharina CM Schuiling-Veninga, Barbara C van Munster, Sumaira Mubarik, Hendrika J Luijendijk, Eelko Hak

**Affiliations:** 1 Groningen Research Institute of Pharmacy, PharmacoTherapy, Epidemiology & Economics, University of Groningen, Groningen, The Netherlands; 2 Department of Geriatric Medicine, University Medical Center of Groningen, University of Groningen, Groningen, The Netherlands; 3 Alzheimer Center Groningen, University Medical Center of Groningen, Groningen, The Netherlands; 4 Department of Epidemiology, University Medical Center Groningen, University of Groningen, Groningen, The Netherlands

**Keywords:** Alzheimer's disease, comorbidities, dementia, multiple dispensing, polypharmacy

## Abstract

**Background:**

Alzheimer's disease (AD) is the most prevalent form of dementia, characterized by amyloid-β plaques and neurofibrillary tangles. With an aging population, both AD and comorbidities are increasingly common. Managing comorbidities often requires multiple medications, leading to polypharmacy, defined as the concurrent use of five or more medications.

**Objective:**

This study aimed to estimate and compare the prevalence of polypharmacy one-year prior AD diagnosis compared to non-AD individuals.

**Methods:** A matched cross-sectional design used data from the IADB.nl prescription database (1994–2021), including individuals aged 65 and older with at least one AD medication prescription within a year. Controls were matched by age and sex at a 9:1 ratio. Analyses were stratified by time period (≤2010 and >2010) and further by sex and age.

**Results:**

4150 AD individuals were included and matched with 37,350 controls. AD individuals had a higher prevalence of polypharmacy compared to controls, ≤ 2010 (OR: 1.15, 95% CI: 1.03–1.29), > 2010 (OR: 1.25, 95% CI: 1.16–1.36). Females with AD had slightly higher odds of polypharmacy than males. The prevalence was consistent across different time periods and age groups, with the highest odds in individuals aged 65–74.

**Conclusions:**

AD individuals in the Netherlands exhibit a significantly higher prevalence of polypharmacy in a year pre-AD diagnosis. The findings highlight the complexity of managing multiple comorbid conditions in AD individuals, emphasizing the need for regular review and optimization of medication regimens and the inclusion of non-pharmacological interventions to minimize adverse outcomes and improve quality of life.

## Introduction

Dementia is a general term for a decline in cognitive function severe enough to interfere with daily life. Alzheimer's disease (AD) is the most common type of dementia, characterized by the accumulation of amyloid-β (Aβ) plaques and neurofibrillary tangles in the brain.^1^ Globally, over 55 million people were living with dementia as of 2020, with AD accounting for 60–70% of cases. This number is projected to nearly double every 20 years, reaching 78 million in 2030 and 139 million by 2050.^2^ In the Netherlands, approximately 280,000 people were living with dementia in 2021, with about 70% of those cases attributed to AD,^3^ and the number is expected to double by 2050.^4^ AD is a leading cause of death in the Netherlands, contributing to 2.7% of all mortalities.^5^ Different risk factors are linked to AD, including age, genes, and comorbidities like diabetes, obesity, cardiovascular diseases, infectious agents, and psychiatric disorders.^6^ Some of these conditions require the administration of multiple medications leading to polypharmacy (≥5 concomitant medications^7^).

The number of adults with polypharmacy has been increasing as well. A study was conducted to compare the prevalence of polypharmacy in older adults between the Netherlands and the United States. A study comparing polypharmacy in older adults found an increase in prevalence from 3% to 8% in the Netherlands (1999–2014) and from 8% to 15% in the US (1999–2012).^8^ While sometimes necessary, polypharmacy increases the risk of adverse reactions, drug interactions, and medication errors.^9^ For example, medications with anticholinergic properties, such as some antidepressants and antipsychotics, can worsen cognitive function and increase the risk of delirium and dementia.^10^ Additionally, benzodiazepines and other sedative-hypnotics can lead to increased confusion, falls, and fractures.^9^

There is a significant knowledge gap regarding the prevalence of polypharmacy in patients with AD in the Netherlands, as current research lacks comprehensive data on medication use patterns in this population. This gap underscores the need for more detailed studies to understand the scope and impact of polypharmacy on patient outcomes and healthcare management. Therefore, we decided to use a large representative Dutch prescription dispensing database to estimate and compare the prevalence of polypharmacy in AD individuals to a comparable reference group.

## Methods

### Design, setting, and participants

In this matched case-control study, we employed a cross-sectional design using the representative and extensively studied prescription dispensing database, IADB.nl, from the University of Groningen from 1994–2021. This database encompasses information on dispensed medications spanning over 25 years and includes data on more than 1,120,000 individuals and over 120 community pharmacies. Registration in the database is independent of health care insurance, age, and sex. Prescription rates within this database have been shown to be representative of the entire Netherlands. Each individual in the database is tracked individually, and prescription records include details about the date of dispensing, the quantity dispensed, dosage regimen, the number of days the prescription is valid, the prescribing doctor, and the medication (ATC code). Date of birth and sex are known, and each patient is assigned a unique anonymous identifier. The medication records for each patient are comprehensive, with the exception of over-the-counter (OTC) medications and medications administered during hospitalization.^11^ We split the data into two groups, ≤ 2010 and >2010, to explore how updates in practice guidelines influence the use of AD medications and examine changes in physician practice patterns over time as a result of these updates.

The University of Groningen IADB.nl community pharmacy dispensing database contains data that is collected in accordance with the Dutch and European guidelines on privacy requirements (GDPR) for handling human data. Approval of the medical ethics committee was not needed nor required for this study. The SQL and R code for data analysis can be shared by emailing the corresponding author.

### Definition of cases and controls

**Figure 1.**
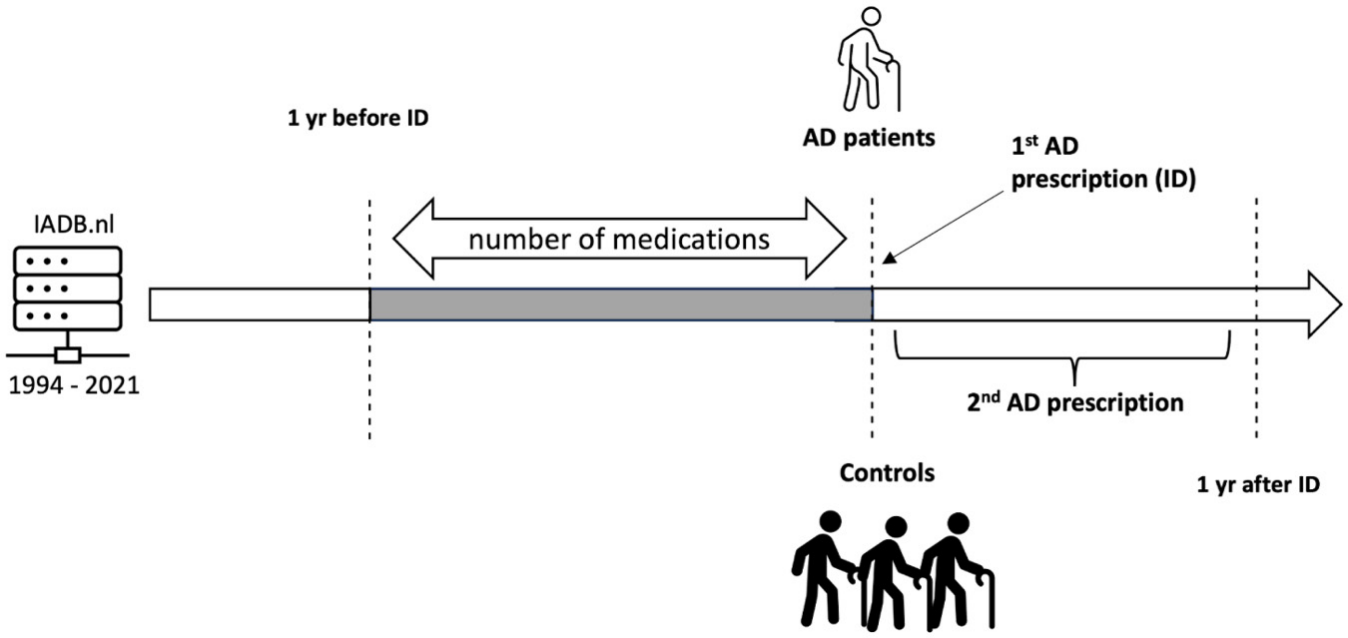
Individuals selection.

We established specific inclusion criteria for AD cases and their sampled controls based on medications that are specifically indicated for AD according to the Dutch guidelines. The case population comprised individuals ≥65 years old with at least one prescription dispensing of the medications for AD (ATC code: “N06D”) in one year, the first prescription dispensing was indicated as the index date (ID). The index date of cases was determined as the date of the first dispensing of AD medication without any AD medication dispensing in the previous year (Figure 1).

Controls were matched with the study population based on age (65 ± 2 years) and sex. We achieved a ratio of 9 controls to 1 case with the same inclusion and exclusion criteria with the exception of disease status.

### Definition of polypharmacy

In this paper, we used the definition of polypharmacy as the simultaneous use of five or more medications, as it is the most commonly used definition.^12,13^ Only medications with at least two prescription dispensing in the 1-year prior the ID were included. The following medication groups were included in the study: antihypertensives (ATC: C02, C03, C07, C08C, C08G, C09), antihyperlipidemia (ATC: C10), cardiovascular medications (ATC: C01, C08D, C08E), antidiabetics (ATC: A10), Proton-pump inhibitors (PPIs) (ATC: A02BC), thyroid medications (ATC: H03AA), asthma/COPD medications (ATC: R03), antithrombotic medications (ATC: B01A), antidepressants (ATC: N06A), bone medications (ATC: M05B), antigout (ATC: M04AA), antiepileptics (ATC: N03AX), antineoplastics (ATC: L01), hormones (ATC: L02), antihistamines (ATC: R06A), calcium supplements (ATC: A12AX), urological medications (ATC: G04), antipsychotics (ATC: N05), anticholinergic medications (ATC: N04A), and antidiarrheal medications (ATC: A07). The fourth ATC level has been considered for this study.

### Statistical analysis

The association between polypharmacy and AD was estimated for periods ≤2010 and >2010. We performed a stratified logistic regression to calculate the odds ratio (OR) and 95% confidence interval (95% CI) comparing cases to controls before and after 2010. The periods were further stratified by sex (male/female) and age categories (65–74, 75–84, and ≥85 years). The medication class prevalence was measured as a percentage in the year prior the index date for both cases and controls. A sensitivity analysis was performed on individuals with two or more medication dispensing to assess the robustness of the findings. This analysis aimed to explore whether the observed trends in polypharmacy prevalence held true when focusing specifically on patients with more frequent medication use. SQL has been used for creating dataset and SPSS version 28 and RStudio version 2023.06.2 + 561 (2023.06.2 + 561) for the analysis.

## Results

### Baseline characteristics

**Figure 2.**
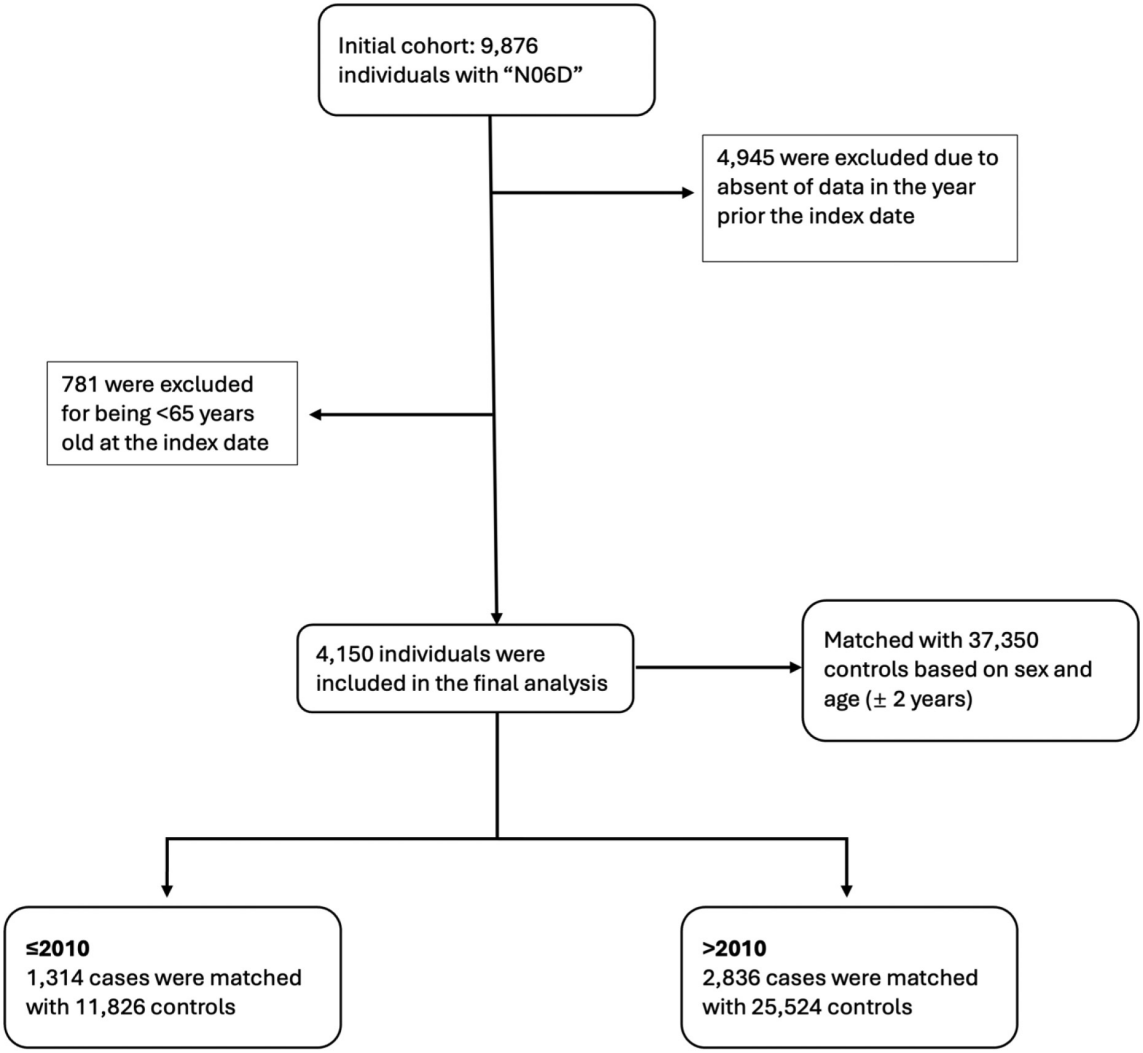
A flow diagram of the exclusion criteria and the number of participants ended up in the study. * This number includes the individuals who received at least one prescription. A sensitivity analysis was also performed including only individuals with 2 or more AD prescriptions.

The initial number of cases was 9876 individuals. After excluding those without records 365 days before the index date, the sample size was reduced to 4931. An additional 781 individuals under age 65 were excluded, leaving 4150 AD cases. Of these, 1314 cases (≤2010) were matched with 11,826 controls, and 2836 cases (>2010) were matched with 25,524 controls (Figure 2). The characteristics of included individuals at the index date showed that 55% of participants from both groups are females ≤2010 while equal number of male and female participants in both groups >2010. For the time period up to 2010, cases were dispensed more medications on average (mean: 5.5) than controls (mean: 4.6). The median number of medications also shows that AD cases had a slightly higher number (median: 5) than controls (median: 4). For the period after 2010, both AD cases and controls had an increase in the mean number of medications compared to the previous period. AD cases now had a mean of 5.8 medications, while controls had a mean of 5. The median number of medications remained the same as in the earlier period (median: 5 for AD cases and 4 for controls). The percentage of individuals claimed AD medications ≤ 2010 (31.7%) is lower than > 2010 (68.3%) (Table 1a, 1b).

**Table 1.** Baseline characteristics: (a) including individuals before 2010; (b) including individuals after 2010.

≤2010		
a)	AD case n = 1314	Controls n = 11,826
Sex	**%**	**%**
Male	45	45
Female	55	55
Age mean	75.6 (6.5)	75.6 (6.5)
Age categories	**%**	**%**
65–74	38.3	40.8
75–84	51.4	48.6
>= 85	10.2	10.5
Mean number of medications	5.5	4.6
Median number of medications	5	4

>2010		
b)	AD cases n = 2836	Controls n = 25,524
Sex	%	%
Male	50.1	50.1
Female	49.9	49.9
Age mean	76.6 (6.7)	76.6 (6.7)
Age categories	**%**	**%**
65–74	33.5	38.4
75–84	51.5	47.6
>= 85	15.0	14.0
Mean number of medications	5.8	5
Median number of medications	5	4

### Prevalence of polypharmacy

In the analysis of data collected prior to 2010, the odds ratio (OR) for the overall association between polypharmacy and AD was 1.15 (95% CI: 1.03–1.29), indicating a modest but statistically significant increase in the odds of polypharmacy among AD cases compared to controls. We then analyze the variation of OR between strata, the OR for males was 1.18 (95% CI: 0.99–1.40) and for females was 1.13 (95% CI: 0.97–1.32). The odds ratio across different age categories were calculated. The OR for the 65–74 age group was 1.21 (95% CI: 0.99–1.46), for the 75–84 age group was 1.11 (95% CI: 0.95–1.31), and for the ≥85 age group was 1.07 (95% CI: 0.75- 1.54). with largely overlapping 95% CI (Table 2a).

**Table 2.** The prevalence of polypharmacy in AD and control groups: (a) include the prevalence of polypharmacy in individuals before 2010; (b) include the prevalence of polypharmacy in individuals after 2010.

≤2010			
a)	AD cases n = 1314	Controls n = 11,826	OR (95%)
	n with polypharmacy / n without polypharmacy	n with polypharmacy / n without polypharmacy	
Overall	552/762	4563/7263	1.15 (1.03–1.29)
Sex			
Male	255/337	2081/3247	1.18 (0.99–1.40)
Female	297/425	2482/4018	1.13 (0.97–1.32)
Age categories			
65–74	176/328	1487/3341	1.21 (0.99–1.46)
75–84	308/368	2467/3284	1.11 (0.95–1.31)
>= 85	68/66	609/638	1.07 (0.75–1.54)

>2010			
b)	AD cases n = 2836	Controls n = 25,524	OR (95%)
	n with polypharmacy / n without polypharmacy	n with polypharmacy / n without polypharmacy	
Overall	1328/1508	10,532/14,992	1.25 (1.16–1.36)
Sex			
Male	638/789	5281/7517	1.15 (1.03–1.28)
Female	690/724	5251/7475	1.36 (1.22–1.51)
Age categories			
65–74	377/572	3207/6594	1.36 (1.18–1.55)
75–84	726/735	5543/6601	1.18 (1.06–1.31)
>= 85	225/201	1782/1797	1.12 (0.92–1.38)

In the data collected after 2010, the overall OR for polypharmacy and AD was higher than in the pre-2010 data, at 1.25 (95% CI: 1.16–1.36). For males, the OR was 1.15 (95% CI: 1.03–1.28), while for females, the OR was 1.36 (95% CI: 1.22–1.51). In age-stratified analyses, the odds ratio in the 65–74 age group was 1.36 (95% CI: 1.18–1.55), 75–84 age group was 1.18, (95% CI: 1.06–1.31), and for individuals over the age of 85, the OR was 1.12 (95% CI: 0.92- 1.38) with largely overlapping 95CI (Table 2b).

We also conducted a sensitivity analysis including individuals with 2 or more prescription dispensing only. The number of cases included was 3660 matched with 32,940 controls. The results showed no difference in terms of the prevalence of polypharmacy when compared to the original cohort (see Supplemental Table 1).

### Prevalence of different medication groups

**Figure 3.**
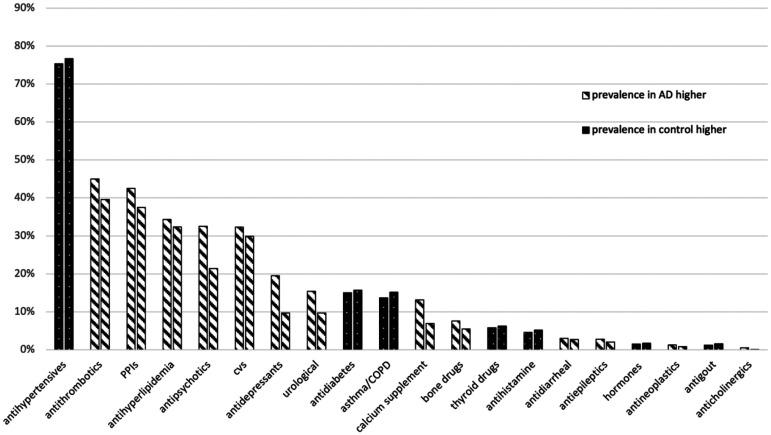
Percentages of different medication groups used in AD and control groups.

Specific medication classes were categorized into different groups based on their indications. The results showed that some medication groups were more frequent in the AD group, while others were more frequent in the control group. The prevalence of the following groups was higher in individuals with AD: antithrombotic, PPIs, antihyperlipidemia, antipsychotics, Cardiovascular system (CVS) medications, antidepressants, urological, calcium supplements, bone medications, antidiarrheal, antiepileptics, and anticholinergics (Figure 3). The figure also illustrates that the prevalence of asthma/COPD and gout medications is lower in individuals with Alzheimer's. Regarding the other groups, no significant differences were found.

## Discussion

The study aimed to investigate the prevalence of polypharmacy among AD individuals and compare it with those without AD, particularly focusing on various medication classes and their potential impact on AD. The results of the study revealed a significantly higher prevalence of polypharmacy among AD individuals compared to the control group. Sex differences were notable, with females having a slightly higher prevalence of polypharmacy compared to males. AD individuals, particularly those aged 65–74, exhibited the highest odds of experiencing polypharmacy. Certain medication classes were more frequently prescribed to AD individuals like PPIs and CNS-active medications.

Our findings are consistent with previous research on polypharmacy among AD individuals. Andersen et al. (2011) identified a significant prevalence of polypharmacy within the AD population, highlighting the complexities of managing multiple comorbid conditions.^11^ The data from Figure 3 in our study reveals a significant insights into medications commonly prescribed to AD individuals compared to control. Antithrombotic medications are often prescribed to prevent blood clots. Their higher prevalence among AD individuals suggests a greater focus on managing cardiovascular risks, which are common in AD population.^14^ This is in line with Wang et al. (2018), who found a high prevalence of cardiovascular diseases in AD individuals.^15^ It can be hypothesized that individuals diagnosed with AD, who also have a history of CVD, are more likely to develop mixed dementia (a combination of AD and vascular dementia). This may be due to the high prevalence of CVD in older populations, particularly among those with AD, which could also explain the frequent use of medications associated with managing both conditions. According to Farnsowrth von Cedarwald et al., cardiovascular risk trajectory predicted an increased risk of developing AD or vascular dementia.^16^

PPIs are also commonly used to reduce gastric acid and are more frequently used prescribed to AD individuals. However, the long-term use can be associated with negative outcomes like increased risk of bone fractures and possibly even cognitive decline.^13^ CNS medications are more prevalent in AD individuals since they are managing neuropsychiatric symptoms like depression and agitation. While beneficial for symptom control, they require careful monitoring due to potential side effects.^12^

The increase in the number of CNS medication might be due to the slow progressive nature of the disease, some psychological symptoms might manifest before the incidence of AD. According to Jost el al., depression, change in mood, and other symptoms might appear 24 months before the diagnosis with AD.^17^

The higher prevalence of polypharmacy in AD individuals can be attributed to the complexity of managing multiple comorbid conditions commonly associated with aging and AD. it is crucial to regularly review and optimize medication regimens for AD individuals to minimize the risks of adverse drug reactions and potential drug-drug interactions. Healthcare providers should prioritize deprescribing medications that might be harmful for AD individuals when possible and consider non-pharmacological interventions to manage symptoms and improve quality of life.

Wang et al., explored the influence of medical comorbidities on medication management in AD individuals, finding that these comorbidities significantly impact the complexity of polypharmacy management. This observation aligns with our study, which underscores the need for meticulous management of multiple medications in AD individuals to address chronic conditions effectively.

### Strengths and limitations

To our knowledge, this study is the first attempt to investigate the prevalence of polypharmacy in AD individuals in the Netherlands, while in Europe, a similar study was conducted but with significantly smaller groups.^18^ This study is the first to examine various medication classes and their potential effects on AD, with the added strength of a large sample size of cases and controls, improving precision. Using data from the IADB.nl prescription database, which collects dispensing records from Dutch pharmacies, participants were included based on prescribed medications (excluding OTC use). However, actual medication usage is unknown. Ethnicity was not included due to the low representation of ethnic minorities in the northern Netherlands, unlike cities like Amsterdam or Rotterdam. The dataset also lacked information on specific diseases, ICD codes, diagnoses, and laboratory results, making it difficult to assess the impact of comorbidities or include other types of dementia. Moreover, important factors such as education level, socioeconomic status, and smoking were not analyzed. While over 280,000 individuals live with dementia in the Netherlands, only 187,000 have been diagnosed and are receiving medical treatment.

Over 100,000 individuals do not seek healthcare support. Some rely on family and friends, while others avoid medical attention due to fear of diagnosis or lack of awareness.^3^ Underdiagnosis of AD could lead to information bias, with undiagnosed individuals mistakenly included as controls. This misclassification may weaken the observed association between AD and polypharmacy, underestimating the true effect. Lang et al. highlighted the prevalence of undetected dementia in the community. The overall results indicated that 61.7% of cases with dementia are undetected around the world and 53% in Europe.^18^ The absence of OTC medication data introduces information bias, as the study may underestimate the true extent of polypharmacy among both AD individuals and controls. Consequently, the study might not fully represent the medication-related risks faced by the elderly population.

### Conclusions

Our study revealed higher prevalence of polypharmacy among AD cases the year prior the first AD medication dispensing compared to those without AD. AD individuals are more likely to be prescribed multiple medications, particularly antithrombotic agents, PPIs, antihyperlipidemia drugs, antipsychotics, and cardiovascular medications. This complexity necessitates regular medication reviews to minimize adverse drug reactions and interactions. This highlights the need for careful monitoring of medication use in older adults with cognitive impairments, as addressing polypharmacy early may improve health outcomes and potentially delay further cognitive decline prior to an AD diagnosis. Future research should address limitations like actual medication adherence and over-the-counter medication use for a comprehensive understanding of medication-related risks in AD management.

## Supplemental Material

sj-docx-1-alz-10.1177_13872877241305799 - Supplemental material for A matched case-control study on polypharmacy and co-medications one year before drug treatment for Alzheimer's disease Supplemental material, sj-docx-1-alz-10.1177_13872877241305799 for A matched case-control study on polypharmacy and co-medications one year before drug treatment for Alzheimer's disease by Ali Alghamdi, Stijn de Vos, Jens HJ Bos, Catharina CM Schuiling-Veninga, Barbara C van Munster, Sumaira Mubarik, Hendrika J Luijendijk and Eelko Hak in Journal of Alzheimer's Disease
