# Supplementary material for: A matched case-control study on polypharmacy and co-medications one year before drug treatment for Alzheimer's disease
Source: J Alzheimers Dis. 2025 Jan 10;103(3):706–13. doi: 10.1177/13872877241305799 (PMC12231824; doi:10.1177/13872877241305799)
Supplement: sj-docx-1-alz-10.1177_13872877241305799 - Supplemental material for A matched case-control study on polypharmacy and co-medications one year before drug treatment for Alzheimer's disease [file sj-docx-1-alz-10.1177_13872877241305799.docx]

**Supplemental Material**

**A matched case-control study on polypharmacy and co-medications one year before drug treatment for Alzheimer’s disease**

**Supplemental Table 1.** A sensitivity analysis on individuals with two or more medication dispensing. The prevalence of polypharmacy in AD and control groups: (a) include the prevalence of polypharmacy in individuals before 2010. (b) include the prevalence of polypharmacy in individuals after 2010.

| **≤2010** | | | |
| --- | --- | --- | --- |
| **(a)** | **AD cases n=1157** | **Controls n=10,413** | **OR (95%)** |
| Overall | 476 / 681 | 4014 / 6399 | 1.11 (0.98 – 1.26) |
| Sex |  |  |  |
| Male | 221 / 301 | 1809 / 2889 | 1.17 (0.98 – 1.41) |
| female | 255 / 380 | 2205 / 3510 | 1.07 (0.9 – 1.26) |
| Age categories |  |  |  |
| 65 – 74 | 151 / 286 | 1314 / 2908 | 1.17 (0.95 – 1.44) |
| 75 – 84 | 267 / 334 | 2162 / 2921 | 1.08 (0.91 – 1.28) |
| >= 85 | 58 / 61 | 538 / 570 | 1.01 (0.69 – 1.47 |

| **>2010** | | | |
| --- | --- | --- | --- |
| **(b)** | **AD cases n=2509** | **Controls n=22,581** | **OR (95%)** |
| Overall | 1186 / 1323 | 9370 / 13,211 | 1.26 (1.16 – 1.37) |
| Sex |  |  |  |
| Male | 564 / 685 | 4652 / 6589 | 1.17 (1.04 – 1.31) |
| female | 622 / 638 | 4718 / 6622 | 1.37 (1.22 – 1.54) |
| Age categories |  |  |  |
| 65 – 74 | 336 / 492 | 2805 / 5749 | 1.40 (1.21 – 1.62) |
| 75 – 84 | 654 / 656 | 5014 / 5892 | 1.17 (1.04 – 1.31 |
| >= 85 | 196 / 175 | 1551 / 1570 | 1.13 (0.91 – 1.41) |
